# Ischemic toe ulcer secondary to Buerger’s disease managed with low-pressure negative pressure wound therapy and a moisture-preserving interface: a case report

**DOI:** 10.1097/RC9.0000000000000528

**Published:** 2026-05-20

**Authors:** Ahmed Abdelaziz Hassan Mohammed, Aung Chan Thar

**Affiliations:** Department of General Surgery, Kulhudhuffushi Regional Hospital, Kulhudhuffushi City, Republic of Maldives

**Keywords:** Buerger’s disease, case report, ischemic ulcer, limb salvage, moisture-preserving interface, negative pressure wound therapy

## Abstract

**Introduction and importance:**

Buerger’s disease (thromboangiitis obliterans) is a non-atherosclerotic inflammatory vasculopathy characterized by distal ischemia and impaired wound healing. Management of ischemic ulcers in affected patients is challenging, and conventional wound-care strategies may fail due to profound microvascular compromise. Despite the patient’s advanced age, the clinical presentation and vascular findings were consistent with Buerger’s disease-like distal ischemic vasculopathy after the exclusion of other causes of distal ischemia.

**Presentation of case:**

We report the case of a 71-year-old male chronic smoker who presented with a painful, non-healing ulcer on the left great toe. Vascular imaging demonstrated preserved proximal arterial patency without hemodynamically significant stenosis, while distal perfusion was reduced. Following extensive surgical debridement, negative pressure wound therapy (NPWT) was initiated. Early marginal necrosis developed when it was applied directly to the wound bed. Subsequent modification of the technique, using low-pressure continuous NPWT (−50 mmHg) combined with a moisture-preserving interface, led to progressive granulation tissue formation, wound contraction, and epithelialization, resulting in sustained wound healing.

**Clinical discussion:**

In ischemic wounds associated with Buerger’s disease, direct application of NPWT may exacerbate tissue injury due to desiccation and microvascular compromise. Introducing a moisture-preserving interface beneath low-pressure NPWT can help maintain tissue hydration, protect fragile ischemic tissue, and optimize the wound microenvironment.

**Conclusion:**

This case highlights the importance of tailoring NPWT techniques to the underlying vascular pathology. Low-pressure NPWT, combined with a moisture-preserving interface, may represent a useful approach for managing ischemic wounds in patients with Buerger’s disease.

## Introduction

Buerger’s disease, also known as thromboangiitis obliterans, is a segmental, non-atherosclerotic inflammatory occlusive vasculopathy that predominantly affects small- and medium-sized arteries and veins of the extremities. The disease is strongly associated with tobacco use and is characterized by inflammatory thrombus formation with relative preservation of the vascular wall. This unique pathology results in profound microvascular dysfunction, leading to distal ischemia despite the absence of significant proximal arterial occlusion^[^1–4^]^.

HIGHLIGHTS

- Distal ischemic ulcer in an elderly chronic smoker, consistent with Buerger’s disease.
- Unprotected negative pressure wound therapy resulted in marginal necrosis in ischemic tissue.
- Low-pressure NPWT (−50 mmHg), combined with a moisture-preserving interface, prevented recurrent necrosis.
- Tailored wound-bed protection enhanced granulation and contributed to limb salvage.

Clinically, patients commonly present with rest pain, ischemic ulcers, or digital gangrene^[^1,2^]^. Wound healing in Buerger’s disease is particularly challenging, as tissue-level perfusion is severely compromised even when macrovascular patency appears preserved on Doppler or angiographic studies. Consequently, ischemic ulcers are often refractory to conventional wound care and prone to recurrent necrosis following debridement^[^5^]^.

Management of advanced Buerger’s disease remains difficult. Absolute smoking cessation is the cornerstone of therapy; however, once tissue loss has occurred, additional interventions are frequently required. Revascularization options are often limited due to the distal and segmental nature of the disease, necessitating alternative strategies focused on infection control, optimization of microcirculation, and promotion of wound healing. NPWT has been shown to enhance granulation tissue formation and reduce edema, but its application in ischemic wounds carries a risk of excessive desiccation and marginal necrosis when used without adequate protection of the wound bed^[^6–9^]^.

Recent approaches emphasize the importance of maintaining a stable, moisture-preserved microenvironment to support cellular viability and prevent further ischemic injury. Combining low-pressure NPWT with a moisture-preserving interface may mitigate these risks by protecting fragile ischemic tissue, enhancing microcirculatory perfusion, and promoting sustained wound healing.

Herein, we report a challenging case of a non-healing great toe ulcer secondary to Buerger’s disease in a chronic smoker, successfully managed using extensive surgical debridement followed by low-pressure NPWT combined with a moisture-preserving interface. This case highlights the role of tailored wound-care strategies in overcoming recurrent necrosis and achieving limb salvage in advanced ischemic disease.

Recent literature has highlighted atypical presentations of Buerger’s disease in elderly patients, expanding the traditional demographic profile of the disease^[^10–12^]^, as well as the challenges of applying NPWT in ischemic wounds. Emerging evidence supports modified NPWT strategies and limb salvage approaches in distal ischemic ulcers.

This case report has been presented in line with the SCARE 2025 criteria^[^13^]^.

## Timeline

The clinical course is summarized in Table 1.

**Table 1:** Clinical timeline.

Time point	Clinical event
1 month prior to presentation	Incision and drainage performed.
Week 0	Presentation to wound care clinic.
Weeks 1–3	Serial surgical debridement with recurrent marginal necrosis.
Week 3	Initiation of negative pressure wound therapy.
Week 4	Modification to low-pressure NPWT with moisture-preserving interface.
8 weeks post-discharge	Sustained wound healing achieved.

## Case presentation

A 71-year-old male farmer, a known case of dyslipidemia and hypertension, presented with a painful, non-healing ulcer over the left great toe. Notably, the patient’s age is atypical for Buerger’s disease; however, his long-standing history of heavy smoking and distal ischemic presentation raised clinical suspicion.

The patient had a long history of heavy smoking since childhood (beedi/hand-rolled cigarettes). The patient denied alcohol consumption. There was no significant family history of vascular or autoimmune disease. He was socially independent and able to perform activities of daily living prior to the onset of the ulcer. He had no drug allergies. His body mass index was 26 kg/m^2.^ The patient had no history of diabetes mellitus.

## History of present illness

Incision and drainage (I&D) were performed 1 month earlier, but the wound failed to heal despite regular dressing. The patient continued to experience severe pain. He was subsequently referred to the wound care clinic for further evaluation and management.

## Clinical examination

**Figure 1.**
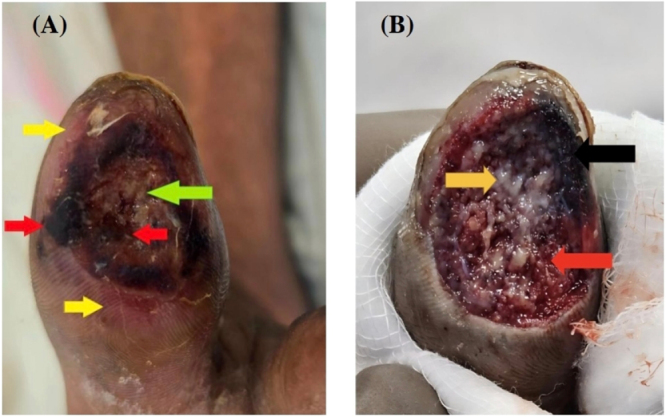
(A) Wound at initial presentation demonstrating necrotic tissue (red arrow), slough within the wound bed (green arrow), and surrounding peri-wound inflammation (yellow arrow). (B) Wound appearance after application of negative pressure wound therapy without a protective interface, demonstrating heterogeneous tissue composition with areas of granulation tissue (red arrow), patchy slough (yellow arrow), and marginal ischemic necrosis (black arrow), highlighting the adverse effect of unprotected NPWT on ischemic tissue.

Wound assessment revealed necrotic wound edges, a wound bed containing slough and necrotic tissue, and surrounding peri-wound inflammation. Multiple surgical debridements were performed (four sessions), each followed by recurrent marginal necrosis (Fig. 1A).

## Investigations

- Laboratory investigations were performed to evaluate infection, metabolic abnormalities, and potential contributing factors.

Complete blood count revealed hemoglobin of 11.3 g/dL, white blood cell count of 9.43 × 10^9^/L, and platelet count of 390 × 10^9^/L. Inflammatory markers showed C-reactive protein of 5 mg/L and erythrocyte sedimentation rate (ESR) of 16 mm/hr. Renal function was within normal limits (serum creatinine 1.1 mg/dL, urea 40 mg/dL). Glycemic assessment demonstrated a fasting blood glucose of 109 mg/dL and HbA1c of 5.4%, excluding diabetes mellitus.

- Doppler ultrasound: Arterial duplex ultrasound of the affected limb demonstrated moderate atherosclerotic changes without hemodynamically significant stenosis. Arterial luminal patency was preserved, with reduced distal flow signals, particularly within the small-caliber vessels, and no evidence of proximal arterial occlusion.

Although Doppler ultrasound findings in Buerger’s disease are often non-specific, it serves as a valuable first-line imaging modality to exclude significant atherosclerotic occlusive disease. In this case, the absence of significant arterial stenosis, despite moderate atherosclerotic changes, supported a non-atherosclerotic etiology when correlated with the clinical findings.

These findings, in conjunction with the patient’s heavy smoking history and distal ischemic symptoms, support the diagnosis of Buerger’s disease.

- CT angiography: CTA provides a detailed assessment of distal arterial involvement in Buerger’s disease, typically demonstrating segmental occlusions and characteristic corkscrew collateral vessels with relative sparing of proximal arteries. CTA is particularly useful in differentiating Buerger’s disease from atherosclerotic peripheral arterial disease and in assessing the extent of vascular involvement.

**Figure 2.**
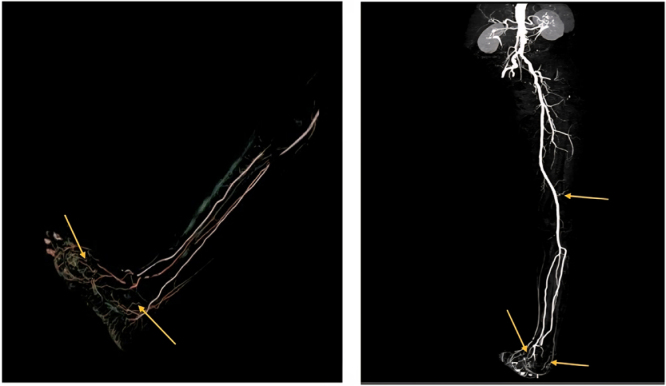
Computed tomography angiography with 3D reconstruction of the left lower limb demonstrating distal collateral vessel formation with relative sparing of the proximal arteries and distal arterial refilling through prominent tortuous corkscrew collateral vessels around the foot (arrows), consistent with microvascular involvement. An angiographic pattern characteristic of thromboangiitis obliterans in the appropriate clinical context.

In our case, CTA showed atherosclerotic vascular changes in the form of scattered calcified and soft plaques causing mild luminal narrowing without acute ischemia or significant stenosis, with an extensive network of tortuous, corkscrew-shaped collateral vessels seen around the ankle and foot (Fig. 2).

The demonstration of tortuous, corkscrew collateral vessels represents a characteristic angiographic finding that strongly supports the diagnosis of thromboangiitis obliterans (Buerger’s disease).

Importantly, the left dorsalis pedis and distal foot arteries supplying the great toe were patent, with normal caliber, confirming preserved distal perfusion.

Although the distal arteries supplying the great toe were patent and of normal caliber, this does not exclude ischemia. In Buerger’s disease, microvascular involvement with inflammatory thrombotic occlusion leads to impaired tissue perfusion at the arteriolar and capillary levels. Therefore, ischemic tissue damage may occur despite preserved large-vessel patency.

- X-ray of the foot: no evidence of osteomyelitis.
- Anti-neutrophil cytoplasmic antibodies (ANCA) and Anti-nuclear antibodies (ANA): negative

## Differential diagnosis

Atherosclerotic peripheral arterial disease, diabetic ischemic ulcer, vasculitis, and embolic arterial occlusion were considered. However, the absence of significant proximal stenosis, negative autoimmune markers, preserved distal perfusion to the great toe, and the presence of characteristic corkscrew collateral vessels favored the diagnosis of thromboangiitis obliterans.

## Diagnosis assessment

Comprehensive diagnostic evaluation was performed to determine the underlying cause of distal ischemia.

Diabetes mellitus was excluded based on normal fasting blood glucose and HbA1c levels.

Significant atherosclerotic peripheral arterial disease was unlikely due to the absence of hemodynamically significant stenosis on Doppler and CT angiography.

Embolic disease was considered less likely in the absence of a proximal embolic source.

Vasculitis was excluded by negative autoimmune markers (ANA and ANCA).

Despite the preserved patency of distal arteries, the presence of ischemic ulceration was attributed to microvascular dysfunction, consistent with Buerger’s disease.

Despite the atypical age, the diagnosis was supported by the presence of characteristic corkscrew collateral vessels, heavy tobacco exposure, distal ischemic presentation, and the exclusion of other etiologies.

## Diagnosis

Based on clinical features, smoking history, and imaging findings, the patient was diagnosed with Buerger’s disease-like distal ischemic vasculopathy.

## Management plan

The management strategy aimed to optimize distal perfusion, control tissue necrosis, and promote wound healing.

### Surgical debridement

Extensive surgical debridement was performed under regional anesthesia in a sterile operating room environment until viable tissue was achieved, with no bone exposure. Standard skin preparation with a chlorhexidine-based antiseptic solution was used. The procedure was performed by a consultant surgeon experienced in advanced wound management, and no intraoperative complications occurred.

Initial debridement was undertaken to remove necrotic tissue and establish a viable wound bed. A second debridement was required following the development of marginal necrosis after the initial application of unprotected negative pressure wound therapy (NPWT). Indications for debridement included the presence of necrotic tissue, slough, and nonviable wound margins.

The lesion was likely initiated by minor trauma to the affected toe and subsequently complicated by a secondary infection, leading to localized abscess formation and progressive tissue necrosis.

### Negative pressure wound therapy

NPWT was initiated in continuous mode at low pressure (−50 mmHg) using a commercially available NPWT system (Confort C300 NPWT therapy unit). Continuous mode was preferred over intermittent therapy to avoid repeated ischemia–reperfusion stress and pain, which may further compromise fragile microcirculation in ischemic tissue^[^14,15^]^.

The initial application was performed without a protective interface. During the first dressing change, which occurred after 48 hours, marginal wound necrosis was observed (Fig. 1B).

Low-pressure NPWT (−50 mmHg) was selected to minimize microvascular compression and ischemic injury in compromised tissue^[^14^]^.

### Moisture-preserving interface

To prevent further tissue desiccation and protect the ischemic wound bed, a moisture-preserving interface was subsequently introduced beneath the NPWT dressing. The interface consisted of:

- Hyaluronic acid–based hydrogel with colloidal silver (MegaHeal®).
- A soft silicone wound contact layer (Mepitel One®).
- Silver-impregnated polyurethane foam dressing under NPWT (Comfort NPWT dressing set).

The hyaluronic acid–based hydrogel was applied directly to the wound bed to maintain a moist environment and support cellular viability. A silicone contact layer was then applied as a protective interface to prevent direct contact between the NPWT foam and the fragile ischemic tissue. Given the chronic nature of the wound and the likelihood of bacterial colonization, silver-containing dressings were incorporated to provide antimicrobial protection and reduce bioburden.

**Figure 3.**
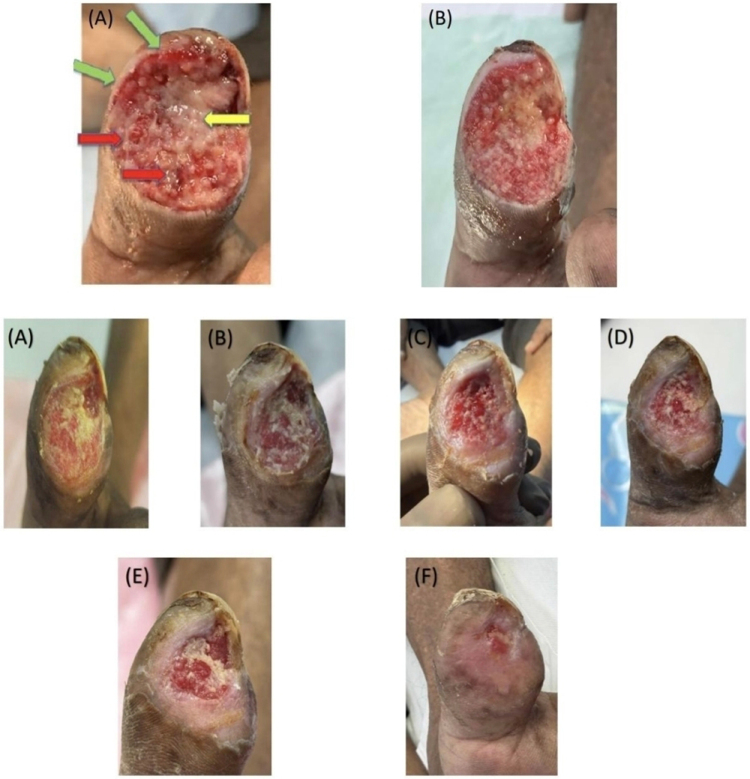
Top row (A–B). Progressive wound improvement following introduction of a moisture-preserving interface under low-pressure NPWT: (A) Early phase demonstrating mixed wound bed with residual slough (yellow arrow), emerging granulation tissue (red arrow), and viable wound edges (green arrow). (B) Continued therapy showing marked granulation tissue proliferation, significant reduction of slough, and a more uniform, healthy wound bed. Bottom rows (A–F). Serial post-discharge wound follow-up photographs demonstrating gradual reduction in wound size with progressive granulation tissue formation and advancement of epithelialization over time: (A) at discharge, (B) 1 week, (C) 2 weeks, (D) 4 weeks, (E) 6 weeks, and (F) 8 weeks post-discharge.

Dressings were changed every 2 days. Following this modification, progressive granulation tissue formation was observed without further marginal necrosis (Fig. 3, top row A–B).

### Adjunctive measures

Prior to surgical debridement and the initiation of NPWT, the patient underwent medical and lifestyle optimization. Smoking cessation counseling was reinforced. The patient was strongly advised to stop smoking, reported significant smoking cessation during treatment, and remained compliant with cessation advice throughout follow-up. Antiplatelet and vasodilatory therapy were initiated to improve peripheral perfusion. Blood pressure and lipid profile were controlled before the intervention (Cilostazol 50 mg twice daily, pentoxifylline 400 mg three times daily, acetylsalicylic acid 75 mg once daily, calcium channel blockers 10 mg three times daily, atorvastatin 20 mg at night time).

## Hospital course and wound progression

The patient remained hospitalized for 1 month. Gradual wound improvement was observed, with a reduction in necrosis, increased granulation tissue, and improved pain control. Granulation tissue became evident within the first week of modified NPWT, followed by progressive wound contraction over the subsequent weeks, with the initiation of epithelialization observed by the third week. Pain severity improved from 9/10 to 2/10 on the visual analog scale. NPWT was continued throughout the admission for 25 days, with dressing changes every 2 days.

The observed wound contraction suggests myofibroblast activity, likely enhanced by NPWT, while the initiation of epithelialization reflects the restoration of a favorable wound microenvironment.

## Complications and adverse events

Early marginal wound necrosis developed following the initial application of NPWT without a protective interface. This complication was managed conservatively with local wound care modification and did not require reoperation. It may be classified as a minor complication (Clavien–Dindo Grade I). No systemic complications, reoperations, or adverse events occurred during hospitalization or follow-up.

## Post-discharge wound follow-up

The patient was discharged after satisfactory local wound improvement and continued wound care on an outpatient basis for 8 weeks. Follow-up assessment demonstrated sustained wound improvement with ongoing contraction and progression of epithelialization, indicating continued response to the applied wound-care strategy (Fig. 3, bottom rows A–F). The patient remained compliant with smoking cessation advice during follow-up.

**Table:** 

Week	Size (cm)
1	4 × 3.5
2	3.8 × 3.2
4	3 × 2.5
6	1 × 1
8	0.5 (largest diameter)

## Intervention adherence and compliance

The patient adhered well to medical therapy and follow-up visits and tolerated low-pressure NPWT without complications. Compliance contributed to sustained wound healing.

## Discussion

Buerger’s disease classically affects younger individuals; however, atypical presentations in elderly patients have been increasingly reported. In such cases, diagnosis requires careful exclusion of atherosclerotic, embolic, and autoimmune causes, with reliance on characteristic clinical features and angiographic findings, particularly the presence of corkscrew collateral vessels.

NPWT is widely utilized to promote granulation tissue formation, reduce edema, and enhance wound contraction^[^6–9^]^. However, its application in ischemic wounds remains controversial. In the setting of compromised microcirculation, conventional NPWT – especially when applied directly to the wound bed – may exacerbate tissue injury through excessive fluid removal, desiccation, mechanical shear stress, and microvascular compression, ultimately leading to marginal necrosis.

In this case, the early development of marginal necrosis following unprotected NPWT highlights the potential risk of standard NPWT application in ischemic tissue. This finding underscores the importance of modifying NPWT parameters according to the underlying vascular pathology.

The use of continuous low-pressure NPWT (−50 mmHg) was specifically selected to minimize microvascular compression and avoid repeated ischemia–reperfusion stress associated with intermittent modes. This approach is supported by emerging evidence suggesting that lower pressures and continuous application may be better tolerated in ischemic or microvascular-compromised wounds^[^16,17^]^.

Furthermore, the introduction of a moisture-preserving interface played a critical role in optimizing the wound microenvironment. Maintenance of adequate moisture is a fundamental principle of wound healing, as it facilitates keratinocyte migration, reduces cellular desiccation, and preserves tissue viability^[^18,19^]^. The hydrogel component provided sustained hydration, while the silicone contact layer acted as a protective barrier against direct foam contact and suction-induced trauma. In addition, the incorporation of silver-containing materials addressed the high likelihood of bacterial colonization in chronic ischemic wounds by reducing bioburden and minimizing the risk of secondary infection^[^5^]^.

Despite preserved patency of distal arteries on imaging, tissue ischemia in Buerger’s disease is primarily driven by microvascular dysfunction. This case highlights that macrovascular patency alone does not guarantee adequate tissue perfusion, and careful assessment of distal perfusion remains essential prior to the application of NPWT.

The favorable progression observed in this case – including early granulation, progressive wound contraction, and epithelialization – suggests that achieving a balance between moisture preservation and controlled negative pressure is essential in preventing recurrent necrosis in ischemic wounds.

Importantly, wound improvement in this case was likely multifactorial, resulting from a combination of complete smoking cessation, repeated surgical debridement, optimized medical therapy, and the modified low-pressure NPWT technique with a moisture-preserving interface.

Overall, this case supports the concept that NPWT should not be applied in a standardized manner in ischemic wounds but rather tailored according to tissue perfusion status and wound characteristics to optimize outcomes and avoid complications.

## Take-home lessons

- In ischemic ulcers associated with Buerger’s disease, unprotected NPWT may precipitate marginal necrosis due to underlying microvascular compromise.
- Careful pre-procedural assessment of distal arterial perfusion is mandatory, as preserved blood supply to the affected digit is a key determinant of surgical success and wound healing.
- Low-pressure continuous NPWT (−50 mmHg) should be considered in ischemic tissue to minimize microcirculatory collapse and excessive desiccation.
- The addition of a moisture-preserving interface can protect fragile wound beds, maintain hydration, and promote sustained granulation and epithelialization.
- Tailoring wound-care strategies to the underlying vascular pathology is essential for successful limb salvage in patients with distal small-vessel disease.

## Strengths and limitations

- Strengths of this report include detailed vascular imaging correlation and clear documentation of wound progression under modified NPWT.
- This report has several limitations. As a single-case study, the findings may not be generalizable. In addition, smoking cessation represents a potential confounding factor, as complete cessation during treatment may have contributed to the observed wound healing. Furthermore, wound improvement in this case was likely multifactorial, influenced by surgical debridement, optimized medical therapy, and the modified NPWT technique. Finally, objective microcirculatory perfusion measurements were not performed, which might have provided additional insight into tissue-level perfusion.

## Conclusion

Low-pressure NPWT combined with a moisture-preserving interface represents an effective and physiologically appropriate strategy for managing ischemic wounds in patients with Buerger’s disease. This tailored approach prevents recurrent marginal necrosis, promotes sustained granulation and epithelialization, and contributes to successful limb preservation. Careful modification of NPWT techniques should be considered in ischemic wounds where microvascular compromise limits conventional healing responses.

## Data Availability

All data generated or analyzed during this case report are included in this published article.

## Artificial intelligence statement

Generative artificial intelligence (ChatGPT, OpenAI, GPT-5.2 model, cloud-based interface, accessed March 2026) was used solely for language refinement, grammatical editing, and improvement of manuscript clarity. No AI tools were used for data generation, clinical decision-making, image interpretation, statistical analysis, or scientific conclusions. All patient-related data were fully anonymized prior to manuscript preparation. All AI-generated suggestions were critically reviewed, verified, and edited by the authors. The authors take full responsibility for the accuracy, integrity, and originality of the content.

## Conference and exclusivity statement

This case has not been presented at any conference or scientific meeting and is not under consideration by any other journal.

## Patient perspective

The patient reported significant pain relief and expressed satisfaction with the outcome and successful limb preservation.
